# The critical role of dietary foliage in maintaining the gut microbiome and metabolome of folivorous sifakas

**DOI:** 10.1038/s41598-018-32759-7

**Published:** 2018-09-27

**Authors:** Lydia K. Greene, Erin A. McKenney, Thomas M. O’Connell, Christine M. Drea

**Affiliations:** 1 University Program in Ecology, Duke University, Durham, NC USA; 2 Department of Evolutionary Anthropology, Duke University, Durham, NC USA; 3 Department of Biology, Duke University, Durham, NC USA; 4 Department of Applied Ecology, North Carolina State University, Raleigh, NC USA; 5 Department of Otolaryngology, Indiana University School of Medicine, Indianapolis, IN USA

## Abstract

The gut microbiome (GMB) of folivores metabolizes dietary fiber into nutrients, including short-chain fatty acids (SCFAs); however, experiments probing the consequences of foliage quality on host GMBs are lacking. We therefore examined GMB structure and function via amplicon sequencing and Nuclear Magnetic Resonance spectroscopy in 31 captive sifakas (*Propithecus coquereli*) during dietary manipulations associated with husbandry. Supplementing standard diets with diverse foliage blends, versus with a single plant species, promoted more diverse GMBs, enriched for taxa implicated in plant-fiber metabolism, but depleted in taxa implicated in starch metabolism and bile tolerance. The consumption of diverse blends was associated with greater concentrations of colonic SCFAs. Abundant foliage, via forest access, promoted compositionally distinct and more stable GMBs, but reduced concentrations of SCFAs, possibly reflecting selection of high-quality leaves. In 11 subjects denied forest access, we examined the temporal pace of microbial shifts when supplemental foliage was abruptly switched between diverse blends and single species. The sifaka GMB responded within days, with community diversity and composition closely tracking foliage diversity. By providing experimental evidence that the folivore GMB is sensitive to minor changes in dietary foliage, we reveal the fragility of specialist GMBs, with implications for managing the wellbeing of endangered wildlife.

## Introduction

Animal gastrointestinal tracts are colonized by communities of microorganisms, known as the gut microbiome (hereafter ‘GMB’), that profoundly impact the health of their hosts^1^. Among the most intuitive and critical functions of the GMB is the promotion of host nutrition: GMBs possess a wealth of metabolic machinery, facilitating pathways that can promote the digestion of various macronutrients^2^, satisfy vitamin requirements^3^ and metabolize ingested tannins^4^ or toxins^5^. Although hosts characterized by all types of feeding strategies rely on their GMBs, the metabolic capacity of the GMB is particularly critical for herbivorous or folivorous animals^6,7^ (i.e., animals that, broadly, consume plant-based diets or, specifically, consume leaf-based diets). This particular reliance owes to ingested plant fiber becoming nutritious only after its conversion, via microbial action, into essential nutrients, such as short-chain fatty acids (SCFAs)^8,9^. Microbially synthesized SCFAs, particularly acetate, propionate and butyrate, nourish host organs and can account for 30–57% of a folivore’s daily energy demands^6^. To extract sufficient nutrients from fibrous diets, herbivores and folivores harbor dense and diverse GMBs that are enriched for microbes and metagenomic pathways associated with plant-fiber metabolism and SCFA production^10–13^.

Given the interdependent relationship between herbivory and the GMB, there has been significant research effort toward understanding factors that either promote GMB symbiosis or lead to GMB imbalance (also known as dysbiosis)^6^. Thus far, however, most wildlife studies have been correlational, linking temporal (primarily seasonal) variation in dietary or macronutrient intake to variation in the GMB. For example, in the herbivorous, North American bison (*Bison bison*), the Tenericutes phylum has been shown to track seasonal consumption of high-protein plants^14^. Likewise, folivorous primates that supplement their typically leafy diets with seasonally available fruit concurrently experience major GMB shifts^10,15–17^, their consortia trading capacity for fiber metabolism and SCFA production with that for sugar and carbohydrate metabolism.

GMB structure and function also have been correlated with variation in the host’s habitat quality, which is potentially a proxy for diet quality. For instance, leaf-eating primates dwelling in primary rainforests host more diverse GMBs than do their peers inhabiting disturbed, fragmented or secondary forests^18,19^. Captivity also shifts the folivore and herbivore GMB, such that, the strong signal of host phylogeny in shaping the GMBs of wild folivores^20,21^ is weaker among captive populations^22^. Moreover relative to their wild counterparts, captive hosts harbor dysbiotic or even ‘humanized’ communities that are strongly linked to sugar-rich, but fiber depleted diets^23,24^. Nevertheless, access to naturalized enclosures and to increased dietary foliage can help alleviate the effects of captivity^23,25^.

It is increasingly clear that variation in dietary plant fiber, whether occurring across seasons, habitats or housing conditions, plays a pivotal role in shaping the herbivore and folivore GMB; however, the paucity of experimental evidence has limited our understanding of the causal linkages between these dietary factors and GMB dynamics. This gap is particularly evident across narrow (i.e., daily and weekly) timescales (although see^4^). We address this deficit using dietary manipulations in the endangered Coquerel’s sifaka (*Propithecus coquereli*).

Endemic to Madagascar, sifakas are an excellent, non-traditional group of primates in which to investigate the effects of foliage quality on the folivore GMB. In the wild, sifakas consume a largely foliage-based diet, comprising hundreds of plant species, as well as seasonally available fruits, seeds, flowers, tree bark and earth^26,27^. Like other folivores, sifakas boast a specialized hindgut gastrointestinal system that includes an enlarged and sacculated caecum, and elongated intestines^28^. Together, these structures stretch to 13–15 times the animal’s body length, requiring a 24–48 hour gut-passage time^29,30^. Similarly specialized, sifaka GMBs are significantly richer and more diverse than are those of frugivorous or omnivorous lemurs^31^ and vary seasonally with fruit availability^16^. Structurally, sifaka GMBs are enriched for microbes that have known cellulose-degrading capabilities^31^; functionally, their consortia have increased capacity for fiber and tannin metabolism, and SCFA production^12^. Compared to non-folivorous lemur GMBs, sifaka GMBs exhibit less inter-individual variation^31^, indicating that they are considerably less flexible and potentially less resilient to perturbation.

Coupling their specialized feeding strategy with their dwindling habitat, sifakas are among the most endangered vertebrates on Earth^32^, their diets increasingly comprising fallback foods that have potentially negative health consequences^33,34^. Sifakas are also notoriously difficult to maintain under captive conditions^35,36^: Currently, the Coquerel’s sifaka is the only sifaka species (out of nine) and the only folivorous lemur species (out of 45) that can be routinely maintained and bred in captivity, outside of Madagascar. Today, the Duke Lemur Center in Durham, North Carolina, maintains the largest captive sifaka breeding population worldwide, where colony success can be partially attributed to the use of local foliage to supplement chow-based diets^37,38^. Greater insight into how foliage supplementation might improve the health and longevity of captive wildlife, potentially via improving host-microbiome symbioses, has empirical value for improving our understanding of folivore biology and applied value for the successful conservation of endangered species.

**Figure 1:**
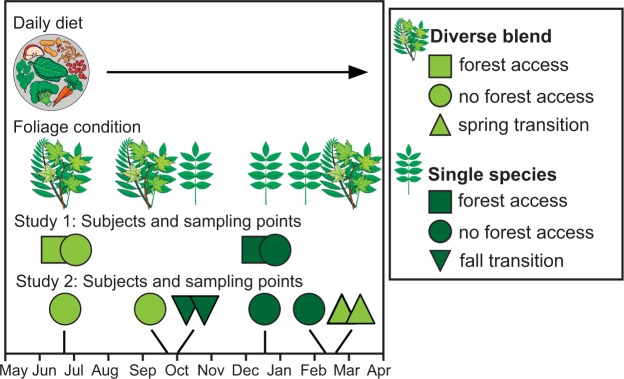
Schematic illustrating the diets received by sifaka subjects relative to the timing of sampling. All of the sifakas received a ‘daily diet’ across the calendar year. This standard fare was supplemented by one of two ‘foliage conditions’, namely a diverse blend in spring through fall or a single species in winter. Study 1 involved all 31 subjects, including those that gained (squares) or were denied (circles) forest access. Faecal sampling occurred once in midsummer and midwinter when all of the animals were provisioned with diverse blends (light green) versus single species (dark green), respectively. Study 2 involved only those 11 subjects that were denied year-round forest access. Sampling occurred an additional three times each in fall and spring, including pre-transition (circles), as well as both 2–4 days and 1 week (triangles) post transitions, when the provisioned foliage abruptly changed between the diverse-blend and single-species conditions. This sampling regimen resulted in two data points for each of the four conditions (diverse blend, fall transition, single species, spring transition). Images provided by S. Bornbusch.

Under the broad hypothesis that variation in host diet underlies variation in both GMB structure and metabolic function, we conducted two experimental studies to probe sifaka GMB dynamics across broad and narrow, temporal scales (Fig. 1). In Study 1, we examined the GMB and colonic metabolome relative to the diversity and abundance of foliage included in the animals’ annual diets: Notably in summer, the standard and balanced, daily sifaka diet is supplemented with a diverse blend of local foliage (hereafter the ‘diverse-blend’ condition), whereas in winter, this same standard diet is supplemented only with winged-sumac (*Rhus copallinum*) (hereafter the ‘single-species’ condition). We consider this change in supplemental foliage as a shift in foliage diversity. Moreover, temperature permitting, some sifakas gain access to large, forested enclosures in which they may semi-free-range and consume additional resources *ad libitum* (hereafter ‘forest access’), whereas other sifakas do not gain forest access (hereafter ‘no forest access’). We consider forest access to reflect foliage abundance. We collected faecal samples from the sifakas in summer and winter, when the animals had consistently been in the diverse-blend or single-species condition, respectively, and when forest access had been maximal or minimal, respectively (Fig. 1). We predicted that sifakas would host the richest, most diverse and most variable gut consortia, enriched for fiber-degrading microbes, when in the diverse-blend condition and when granted forest access. We likewise expected foliage diversity and forest access to be associated with an increase in colonic SCFAs, notably acetate, propionate and butyrate.

In Study 2, we examined GMB dynamics during narrower (week-long), transitional periods, both in the fall, when diverse blends were abruptly replaced with single-species supplements (hereafter ‘fall transition’), and in the spring, when diverse blends were abruptly reintroduced (hereafter ‘spring transition’). We collected samples 1–2 days prior to each dietary switch, as well as 2–4 days and 1 week after each dietary switch, thereby producing two sampling time points for each of the four conditions (diverse blend, fall transition, single species, spring transition; Fig. 1). For sifakas denied year-round, forest access, which controls for dietary variability, we expected GMB structure to track the foliage component of the diet, such that microbial community richness, diversity, variability and capacity for fiber metabolism would change in step with the loss or gain of foliage diversity.

## Results

### Study 1: Linking foliage diversity and abundance to the structure and function of folivore GMBs

**Figure 2:**
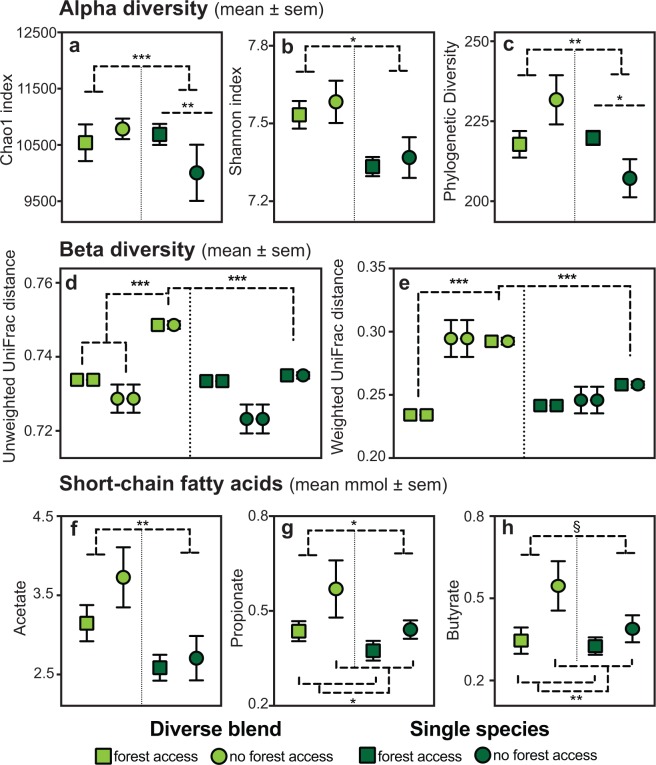
Diversity and short-chain fatty acid (SCFA) concentrations in the sifaka gut microbiome and metabolome relative to foliage diversity and forest access in Study 1. Pictured are measures of alpha diversity, including the (**a**) Chao1, (**b**) Shannon and (**c**) Phylogenetic Diversity indices, beta diversity, as captured by (**d**) unweighted and (**e**) weighted UniFrac distances for all pairwise comparisons (shown by paired symbols), and SCFA concentrations, including (**f**) acetate, (**g**) propionate and (**h**) butyrate. Each measure is graphed relative to the sifakas’ foliage condition, including diverse blends (light green) and single species (dark green), and forest access, including those sifakas that gained forest access at any point during the study (squares), and those that were denied forest access throughout the study (circles). *p < 0.05; **p < 0.01; ***p < 0.001.

Independent of forest access, the sifakas hosted significantly richer and more diverse GMBs when in the diverse-blend condition than when in the single-species condition (Table 1 and Fig. 2a–c). As revealed by Linear Mixed Models (LMM), this finding held across all measures of alpha diversity, namely the Chao1, Shannon and Faith’s Phylogenetic Diversity (PD) indices that, respectively, capture GMB richness, community evenness and microbial phylogenetic representation^39^. Although we found no main effect of forest access on GMB alpha diversity, for two of the indices, Chao1 and PD, there was a significant interaction between foliage condition and forest access. Notably, when diets included single-species supplements and forest access was minimal, sifakas that had gained forest access at any point during the study maintained greater GMB diversity than did peers routinely denied forest access.

**Table 1:** Alpha diversity measures of the sifaka gut microbiome relative to dietary foliage and forest access in Study 1.

Effect	Trend	Chao1 Index		Shannon Index		Phylogenetic Diversity	
		z	*p*	z	*p*	z	*p*
foliage	diverse blend > single species	**3.45**	**<0.001**	**1.99**	**0.046**	**2.88**	**0.004**
forest access	no effect	0.34	0.73	0.51	0.61	1.31	0.19
foliage* forest access	yes > no on single species	**2.95**	**0.003**	0.13	0.90	**2.15**	**0.032**

Note. Significant findings are bolded.

Using unweighted and weighted UniFrac distance measures of beta diversity, which respectively capture the similarity of microbial consortia membership and abundance across pairs of samples^40^, and Bonferonni-corrected student’s *t*-tests, we found that microbial membership differed between pairs of sifakas depending on foliage condition and forest access (Fig. 2d,e). When diets were supplemented with diverse blends and forest access was most consistent, GMBs were less similar (i.e., more variable) for pairs of sifakas in which one member gained and one member was denied forest access, than for pairs in which both members either gained (unweighted: *t*_58_ = −9.32, *p* < 0.001; weighted: *t*_58_ = −14.89, *p* < 0.001) or were denied (unweighted: *t*_58_ = −5.87, *p* < 0.001) forest access. Nevertheless, when diets were supplemented with single species and forest access was most limited, the differences that had been evident between GMBs from hosts with differential forest access disappeared (unweighted: *t*_58_ = −9.99, *p* = <0.001; weighted: *t*_58_ = −9.55, *p* < 0.001).

When in the diverse-blend condition (relative to the single-species condition), the sifakas’ colonic metabolomes had significantly greater concentrations of SCFAs, including acetate and propionate, and modestly greater concentrations of butyrate, as revealed by LMMs (Table 2 and Fig. 2f–h). Forest access had no relation to acetate concentration, but was significantly and negatively associated with both propionate and butyrate concentrations. We found no interaction between foliage condition and forest access for any SCFA.

**Table 2:** Sifaka colonic short-chain fatty acid concentrations relative to dietary foliage and forest access in Study 1.

Effect	Trend	Acetate		Propionate		Butyrate	
		z	*p*	z	*p*	z	*p*
foliage	diverse blend > single species	**3.65**	**0.004**	**2.30**	**0.022**	*1.93*	*0.053*
forest access	no > yes	1.54	0.12	**2.17**	**0.030**	**2.70**	**0.007**
foliage*forest access	no effect	1.03	0.30	0.88	0.38	1.37	0.17

Note. Significant findings are bolded and trends are italicized.

**Figure 3:**
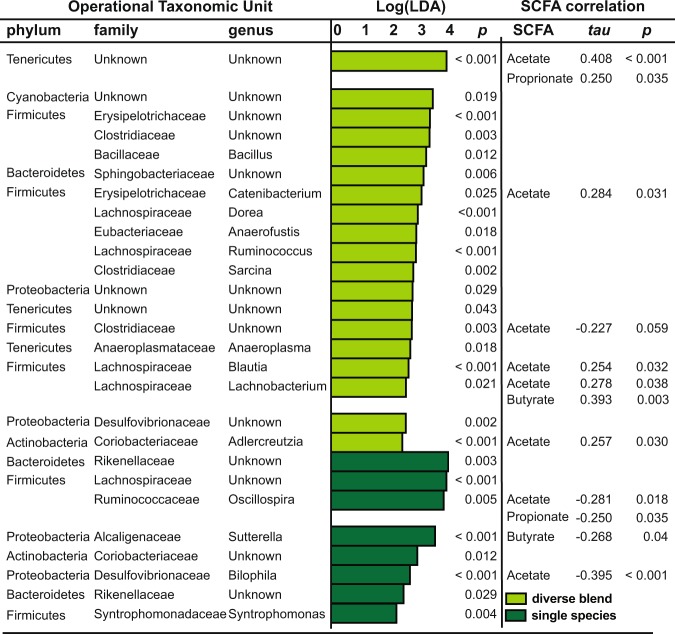
Microbial taxa in the sifaka gut microbiome, at the phylum, family and genus levels, that are significantly enriched relative to foliage condition in Study 1. Shown are taxa for diverse blends (light green) and single species (dark green) that are correlated to short-chain fatty acid (SCFA) concentrations, including acetate, propionate and butyrate.

Overall, the sifaka GMB was dominated by members of the Bacteroidetes and Firmicutes phyla, with smaller contributions from Cyanobacteria, Proteobacteria, and Tenericutes (see Supplementary Material S1), and the abundance of specific microbial genera varied with foliage diversity. An analysis using Linear Discriminant Analysis Effect Size (LEfSe)^41^ revealed that 28 microbial genera (i.e., OTUs) varied with the sifakas’ access to diverse blends versus single species of foliage (Fig. 3), 18 of which remained significant, or trending towards significance, after applying a correction factor for multiple testing (see Supplementary Material S2). Whereas many members of the Clostridiales order and Lachnospiraceae family were enriched when sifakas received diverse blends, Oscillospira, Rikenellaceae members and *Bilophila* were notably enriched when sifakas received the single species. Many of these enriched genera were also those that co-varied with SCFA concentrations (Fig. 3). Overall, correlation analysis indicated that SCFA concentrations co-varied with 12 microbial genera, regardless of foliage diversity (see Supplementary Material S3). Notably, acetate correlated positively with Lachnospiraceae members, such as *Blautia* and *Lachnobacterium*, but negatively with *Oscillospira, Bilophila* and *Prevotella* (Kendall’s *tau* = −0.284, *p* = 0.016). Propionate was likewise negatively correlated with *Oscillospira*, whereas butyrate was negatively correlated with *Sutterella*, but positively correlated with *Lachnobacterium*.

### Study 2: The effect on the GMB structure of transitioning foliage diversity

**Figure 4:**
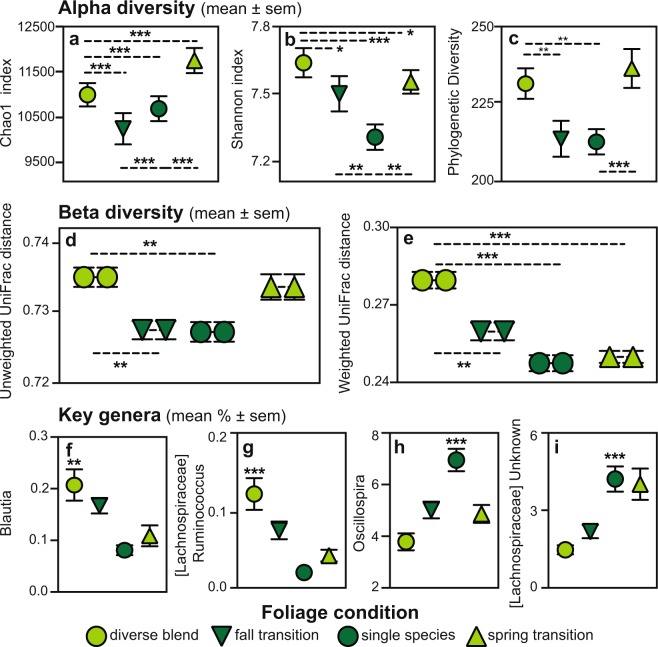
Gut microbiome diversity and the percentages of key microbial genera in the sifakas of Study 2. Pictured are measures of alpha diversity, including the (**a**) Chao1, (**b**) Shannon and (**c**) Phylogenetic Diversity indices, beta diversity, including (**d**) unweighted and (**e**) weighted UniFrac distances (shown by paired symbols), and taxa, including (**f**) *Blautia*, (**g**) *Ruminococcus* from the Lachnospiraceae family, (**h**) *Oscillospira* and (**i**) and an unknown genus from the Lachnospiraceae family. Each measure is binned relative to foliage condition, including diverse blends (light green) and single species (dark green), as well as the timing of sampling, including during periods of consistent foliage supplements (circles), and during the week-long transitions when foliage condition was abruptly switched in fall (downward-pointing triangles) and spring (upward pointing triangles). *p < 0.05; **p < 0.01; ***p < 0.001.

We found that the sifaka GMB responded within mere days to abrupt dietary manipulations. By collapsing the ‘duplicate’ sampling points in each of the four dietary conditions (diverse blend, fall transition, single species, spring transition; Fig. 1), we found that alpha diversity decreased with reduced foliage diversity and was regenerated with increased foliage diversity (Table 3 and Fig. 4a–c). Nevertheless, the magnitude of change varied with each diversity metric: Notably, richness was more extreme during transitional periods (i.e., lowest and greatest during fall and spring transitions, respectively) than during periods of consistent foliage supplements (Fig. 4a); evenness was greatest when animals received diverse blends, was intermediate during transitions, and was lowest when animals received single-species supplements (Fig. 4b); and phylogenetic diversity during each transitional period converged to match that of its respective foliage-diversity condition during more consistent periods (Fig. 4c). Consequently, hosts consuming the same diversity of foliage, whether for two days or several months, harbored comparably diverse consortia.

**Table 3:** Alpha diversity measures of the sifaka gut microbiome relative to dietary foliage across the four experimental periods of Study 2.

Comparison	Chao1 Index		Shannon Index		Phylogenetic Diversity	
	z	*p*	z	*p*	z	*p*
diverse blend vs. single species	**−2.58**	**0.009**	**−5.0**	**<0.001**	**−2.75**	**0.006**
diverse blend vs. fall transition	**−6.08**	**<0.001**	**−1.97**	**0.049**	**−2.6**	**0.009**
diverse blend vs. spring transition	**5.49**	**<0.001**	**−2.25**	**0.025**	0.49	0.63
single species vs. fall transition	**−3.47**	**<0.001**	**3.07**	**0.002**	0.16	0.87
single species vs. spring transition	**8.28**	**<0.001**	**2.98**	**0.003**	**3.38**	**<0.001**

Note. Significant findings are bolded.

Analysis of weighted and unweighted UniFrac distances via Bonferonni-corrected student’s *t*-tests also revealed that sifaka GMBs responded to dietary manipulations (Fig. 4d,e). As captured by pairwise comparisons, variation in both taxonomic representation and abundance between individuals was significantly greater when sifakas were in the diverse-blend condition compared to (1) the fall transition (unweighted: *t*_88_ = 3.98, *p* = 0.005, Fig. 4d; weighted: *t*_88_ = 4.23, *p* = 0.002, Fig. 4e) and (2) the single-species condition (unweighted: *t*_88_ = 3.98, *p* = 0.005, Fig. 4d; weighted: *t*_88_ = 7.17, *p* < 0.001; Fig. 4e). We failed to find a difference between the fall transition and the subsequent single-species condition, indicating that the loss of inter-individual variation between the diverse-blend and single-species conditions occurred within just one week of changing dietary foliage diversity. Likewise, this inter-individual variation in taxonomic representation re-emerged within just one week of diverse blends being reintroduced, such that we also failed to find a difference between the spring transition and the diverse-blend condition (unweighted: *t*_88_ = 0.55, *p* = 1.0). With regard to microbial taxonomic abundance (rather than taxonomic representation), variation between individuals remained low during the spring transition relative to when the sifakas received diverse blends (weighted: *t*_88_ = −7.67, *p* < 0.001).

Consistent with Study 1, the sifaka GMB across Study 2 was dominated by the same microbial genera within the Firmicutes, Bacteroidetes, Cyanobacteria, Proteobacteria and Tenericutes phyla, as well as by the *Akkermansia* genus from the Verrucomicrobia phylum (see Supplementary Material S1), and specific genera of microbes responded rapidly to dietary manipulations. Notably, LEfSe identified 7 taxa that were significantly enriched at particular sampling time points (see Supplementary Material S2). The taxa that were enriched when diets were consistently supplemented with diverse blends (compared to all other dietary conditions) included Lachnospiraceae members, like *Blautia* (Log(LDA) = 3.08, *p* = 0.004, Fig. 4f), *Dorea* (Log(LDA) = 3.27, *p* < 0.001) and *Ruminococcus* (Log(LDA) = 3.46, *p* < 0.001, Fig. 4g), and unknown genera within the Clostridiales order (Log(LDA) = 2.95, p = 0.002) and the Alphaproteobacteria class (Log(LDA) = 2.91, *p* = 0.007). In contrast, the taxa that were significantly enriched when diets were consistently supplemented with the single species (compared to all other dietary conditions) included *Oscillospira* (Log(LDA) = 4.16, *p* < 0.001, Fig. 4h) and an unknown genus within the Lachnospiraceae family (Log(LDA) = 4.15, *p* < 0.001, Fig. 4i). No microbial taxon was enriched during the transition periods. Four of these taxa (*Dorea*, *Ruminococcus, Oscillospira*, and the unknown Clostridiales OTU) remained significant after further correcting for multiple testing (see Supplementary Material S2).

**Figure 5:**
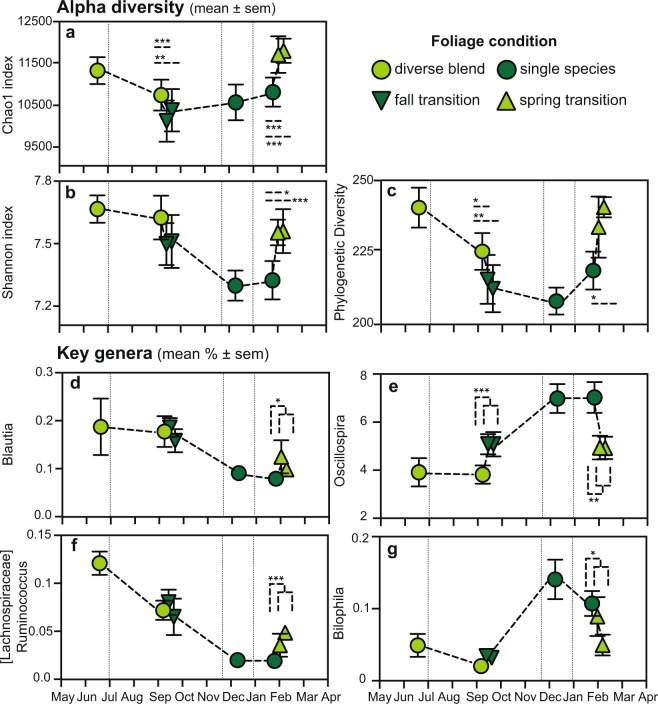
Alpha diversity and the percentages of key microbial genera in the sifaka gut microbiome, relative to each sampling point in Study 2. Pictured here are measures of alpha diversity, including the (**a**) Chao1, (**b**) Shannon and (**c**) Phylogenetic Diversity indices, as well as (**d**) *Blautia*, (**e**) *Oscillospira*, (**f**) *Ruminococcus* from the Lachnospiraceae family and (**g**) *Bilophila*. Each measure is graphed relative to foliage condition, including diverse blends (light green) and single species (dark green), as well as the timing of sampling, including during periods of consistent foliage supplements (circles), and during the week-long transitions when foliage condition was abruptly switched in fall (downward-pointing triangles) and spring (upward pointing triangles). *p < 0.05; **p < 0.01; ***p < 0.001.

When considering only the fall or spring transitions, alpha diversity varied significantly in the predicted direction (Table 4 and Fig. 5a–c). Notably, compared to samples collected immediately prior to the fall transition (when the sifakas received diverse blends), those collected 2–4 days afterwards had significantly lower richness and diversity (Fig. 5a,c). The change in PD became even more pronounced one week after the fall transition (Fig. 5c). Although similar changes were evidenced by the Shannon index, they failed to reach statistical significance (Fig. 5b). Compared to the samples collected immediately prior to the spring transition (when the sifakas received single-species supplements), those collected 2–4 days afterwards were significantly greater in their richness and evenness, with the difference becoming even greater after one week (Fig. 5a,b). The increase in PD during the spring transition reached statistical significance at the one-week sampling point (Fig. 5c).

**Table 4:** Alpha diversity measures of the sifaka gut microbiome relative to dietary foliage across the week-long transitions of Study 2.

Season	Comparison	Chao1 Index		Shannon Index		Phylogenetic Diversity	
		z	*p*	z	*p*	z	*p*
fall transition	pre-fall vs. 2–4 days post	**−4.17**	**<0.001**	−1.56	0.12	**−1.99**	**0.047**
	pre-fall vs. 1-week post	**−2.63**	**0.009**	−1.45	0.15	**−2.79**	**0.005**
spring transition	pre-spring vs. 2–4 days post	**4.73**	**<0.001**	**2.24**	**0.025**	0.257	0.13
	pre-spring vs. 1-week post	**5.54**	**<0.001**	**3.44**	**<0.001**	**1.99**	**0.046**

Note. Significant findings are bolded.

Specific microbial genera also varied significantly within the week-long fall and spring transitions (Fig. 5d–g). LEfSe identified two unknown taxa from the Enterobacteriaceae family that were significantly enriched in samples collected immediately prior to the fall transition, (Log(LDA) > 2.84, *p* < 0.02 for both), whereas immediately after the fall transition, LEfSe identified seven genera that were enriched, including *Oscillospira* (Log(LDA) = 3.90, *p* = 0.001, Fig. 5e), *Prevotella* (Log(LDA) = 4.36, *p* < 0.001) and unknown genera within the Ruminococcaceae (Log(LDA) = 2.88, *p* = 0.009), Lachnospiraceae (Log(LDA) = 3.66, *p* = 0.014) and Coriobacteriaceae families (Log(LDA) = 2.83, *p* = 0.018; see Supplementary Material S2). Likewise, seven taxa were enriched in samples collected immediately prior to the spring transition, including *Oscillospira* (Log(LDA) = 4.03, *p* = 0.005, Fig. 5e), *Phascolarctobacterium* (Log(LDA) = 2.89, *p* = 0.026), *Clostridium* (Log(LDA) = 3.80, *p* = 0.021) and *Bilophila* (Log(LDA) = 2.39, *p* = 0.044, Fig. 5g), whereas another six taxa were enriched immediately after the spring transition, including *Blautia* (Log(LDA) = 2.51, *p* = 0.041, Fig. 5d) and *Ruminococcus* (Log(LDA) = 2.47, *p* = 0.001, Fig. 5f) from the Lachnospiraceae family, *Adlercreutzia* (Log(LDA) = 2.45, *p* < 0.001), and unknown genera from the Mogibacteriaceae (Log(LDA) = 2.80, *p* = 0.008) and Desulfovibrionaceae (Log(LDA) = 2.38, *p* = 0.022) families (see Supplementary Material S2). No taxa remained significant across either transitional period after further correcting for multiple testing (see Supplementary Material S2).

## Discussion

Identifying the nutritional requirements that support the symbiosis between folivores and their GMBs is a burgeoning research aim within conservation and microbial ecology. Here, we contribute to this endeavor by showing that the diversity, variability, membership and function of the folivore GMB change dramatically with even minor shifts in dietary foliage. In omnivores, altering the GMB typically requires extreme dietary perturbation, including experimentally pushing the diets of human volunteers or laboratory rodents to the limits of health^42,43^. Yet, for folivores and herbivores, their complex and specialized gastrointestinal systems may promote the establishment of microbial communities that are susceptible to even small-scale perturbation. Thus, feeding strategy, reflecting the host’s degree of dietary specialization versus generalization, may ‘set up’ the relative resilience or intransigence of the host’s GMB to dietary shifts. Recognizing the relationships between feeding strategy and GMB integrity has implications for our understanding of how microbes can drive vertebrate evolution, including niche specialization and adaptive radiation.

Overall, we found that the diversity of dietary foliage was strongly associated with the diversity of gut consortia. Compared to sifakas that consumed single species, sifakas that consumed diverse blends of foliage exhibited GMBs that were richer, showed greater evenness and had greater representation from diverse microbial lineages, as captured by the Chao1, Shannon and PD indices. Contrary to our expectations, forest access and, thus, the opportunity to forage freely and perhaps select a diverse or high-quality diet did not produce more diverse GMBs; nevertheless, the significant interaction between forest access and foliage diversity suggests that those sifakas that had even minimal forest access better maintained representation from more microbial genera and more phylogenetic lineages as their diets lost foliage diversity. In ecosystem and microbial ecology, community diversity is generally seen as beneficial because it underlies stability by providing multiple or redundant functions^44^. Supporting this ‘diversity begets stability’ framework, the GMBs hosted by sifakas that had forest access were also those that, across the period of study, exhibited the most stability (or least variability) in the various diversity metrics. These results indicate that dietary diversity may indeed underlie GMB stability in this system.

Although the links between dietary quality and GMB diversity in Study 1 occurred across a broader, seasonal scale and could have been correlational, the results from the more narrowly focused Study 2 suggest causality in this relationship. Notably, in our second dietary manipulation, the transitions from the diverse foliage blends of summer to the foliage-limited diets of winter, and vice versa, were abrupt, with the shifts in sifaka GMBs being equally swift. Within a mere two days, a period corresponding to the sifaka’s gut-retention time^30^, GMBs began to converge on the stable state that characterized the sifakas during the following season: In the fall, when diverse blends were substituted with a single species, GMBs immediately lost diversity, whereas in the spring, when diverse blends were reintroduced, GMB diversity was rapidly regenerated.

The different diversity measures revealed how community structure changed during the week-long transitions: Community richness fluctuated somewhat dramatically, whereas phylogenetic breadth more immediately converged onto the following stable state. These results indicate that the loss of foliage diversity in the fall resulted in GMBs that lost representation of specific microbial genera and, indeed, entire lineages. Yet, in the spring, the reintroduction of foliage diversity caused sifaka GMBs to recuperate these microbes, suggesting that these taxa either derive from the plants themselves or potentially persist in low (i.e., methodologically undetectable) abundances until their preferred metabolic substrates are reintroduced. Compared to community richness and phylogenetic diversity, community evenness exhibited a slower adjustment during the transitions, suggesting that whereas the presence of specific microbes immediately shift in response to dietary manipulations, the relative abundances of these taxa require longer than one week to settle into a climax community.

Microbial membership likewise varied with dietary foliage. When the subjects of Study 1 consumed diverse blends, their GMBs were enriched for many Clostridiales taxa, predominantly within the Lachnospiraceae family. These microbes included the *Blautia, Dorea, Lachnobacterium* and *Ruminococcus* genera that are known for their metabolic capacity to ferment plant fibers into SCFAs^13,45,46^. These taxa were also among those that were significantly and positively correlated to SCFA concentrations, further indicating their functional contribution to host energetics^10,47^. In contrast, when foliage diversity was limited, sifaka GMBs were more suited to starch and protein metabolism, as well as to fat and bile tolerance, as evidenced by the increase in *Oscillospira, Bilophila* and members of the Rikenellaceae family^48–50^. These genera were generally negatively correlated to SCFA concentrations, further indicating a tradeoff in plant-fiber (i.e., cellulose) metabolism. Many of the key microbial genera detected in Study 1 were also those that significantly and immediately gained or lost dominance when we transitioned foliage diversity in Study 2: Whereas the abundance of fiber-specializing Lachnospiraceae became more abundant with increasing foliage diversity, microbes like *Oscillospira, Prevotella and Bilophila* concurrently lost abundance. Importantly, the dietary substrates used in microbial starch, protein and fat metabolism were always included in the daily diets of captive sifakas; however, during periods of increased foliage consumption, the microbes specializing on these macronutrients may have been outnumbered and outcompeted by fiber specialists.

Microbial composition, as captured by UniFrac distances, was also associated with the sifakas’ access to forested enclosures, a result that is consistent with our predictions based on previous research^23^. During cold spells, when forest access was restricted, the sifakas hosted a GMB that was relatively homogenized across individuals. By contrast, in summer, those subjects granted forest access harbored distinct consortia from those subjects that were denied forest access. Contrary to our expectations, however, the GMBs of the former, forest-access subjects were associated with lower concentrations of propionate and butyrate than were those of the latter subjects denied forest access. Differences in leaf maturity between our subjects’ diets may help explain the patterns of SCFA production observed. For instance, subjects that were granted forest access may have preferentially selected young, tender and immature leaves^38^ that have low fiber and tannin content, but high sugar and protein content^51–53^, thereby limiting the need for fermentative metabolism, which in turn limited SCFA production. By contrast, animals denied forest access were typically fed mature browse^37^, the metabolism of which requires significant microbial fermentation and may produce SCFAs as byproducts. The microbial metabolism of low-quality leaves into SCFAs that can fulfill energy demands has been suggested as a coping mechanism for folivorous primates during periods of resource scarcity or enhanced resource requirement^10^. Therefore, SCFA production may be one means whereby sifakas that are limited to consuming low-quality, mature leaves meet their energetic demands.

Seasonal variation in the GMB has been shown to occur in wild folivores and herbivores, for which the membership and function of gut consortia reflect seasonally available, young leaves, fruits and proteins^10,14–17^. As in prior studies, we likewise sampled sifakas across seasons^16^; however, the majority of the dietary variation in our study was correlated to, but did not result from, seasonality. Our sifakas always had access to a standard, balanced and complete diet that included chow optimized for leaf eaters, starch-rich potatoes and corn, protein and fat-rich nuts and beans, as well as fiber and vitamin-rich domesticated greens and vegetables. We manipulated only the diversity or abundance of the wild plant species supplementing this standard fare. The strength of our study stems from this direct manipulation of diet and forest access and, thus, our ability to infer causality in host-microbe interaction. Although the benefits of working with captive animals include control in experimental design and feasibility of sampling, researchers have primarily explored the microbial consequences associated with life in captivity versus life in the wild^23,54^. Dietary manipulations that causally probe the GMB of captive wildlife are rare, but could significantly advance our understanding of diet-induced regulation of GMBs across diverse host systems.

In addition to the empirical value of studying and manipulating host-microbe symbioses, research on the GMBs of endangered wildlife is also poised to make significant contributions to conservation and husbandry strategies^55^. Relative to hearty omnivorous hosts, folivorous and herbivorous hosts, like sifakas, face more tenuous survival in the wild^32^ and in captivity^35,36,56^, potentially because of their more ‘fragile’ GMBs; folivores and herbivores are likely more susceptible and, thus, less resistant to dietary and habitat perturbation. Indeed, that feeding strategy may dictate GMB flexibility, resilience or fragility provides a potential mechanism for predicting how wild populations may cope with increasing dietary, habitat or climate change. For the Coquerel’s sifaka, specifically, future studies comparing the GMBs of wild and captive populations should be a research priority. With regard to animal husbandry, more broadly, we suggest that facilities housing folivorous species promote access to forested enclosures whenever possible or prioritize access to a diversity of dietary foliage year round.

## Materials and Methods

### Subjects and housing

The subjects included 31 healthy Coquerel’s sifakas (18 females, 13 males) housed in 10 social groups at the Duke Lemur Center (DLC) in Durham, NC. The subjects ranged in age from 6 months to 23 years: Although our youngest subjects (<4 years) may not have been sexually mature at the time of sampling^36^, sifaka GMBs converge on the adult host’s profile prior to 6 months of age^31^.

The subjects received a once-daily diet, comprising folivore chow (Mazuri Leaf-Eater Primate Diet Mini-Biscuit, No. 5672), nuts or beans, sweet potato or corn, vegetables and kale or collard greens. This standard diet was supplemented with local foliage, harvested from nearby forests and rinsed in 10% bleach. From spring through fall, the sifakas received a diverse blend of fresh foliage that contained minimally winged sumac (*Rhus copallinum*), red bud (*Cercis canadensis*), tulip poplar (*Liriodendron tulipifera*), mimosa (*Albizia julibrissin*), sweet gum (*Liquidambar styraciflua*) and grapevine (*Vitis spp*.). In winter, the sifakas received only defrosted sumac, harvested and frozen during the previous summer. The nutritional content of the plant species fed to the sifakas is presented in the Supplemental Material (S4). The transitions between summer’s ‘diverse blends’ and winter’s ‘single species’ occurred abruptly during fall and spring, with the precise timing depending on the availability of local foliage. Water was always freely available.

Each sifaka group habitually occupied an indoor/outdoor pen (146 m^2^/animal), year-round. Six groups additionally gained forest access (0.6–5.8 ha) when ambient temperatures remained reliably above 5 °C. Warmer temperatures in recent winters resulted in the sifakas gaining forest access intermittently throughout the winter of our study. A seventh group gained forest access only in the summer. The remaining three groups were always denied forest access. With forest access, subjects consumed additional resources *ad libitum*^38^, and, anecdotally, were seen eating various leaves, poison ivy (*Toxicodendron radicans*) and oak (*T. diversilobum*), grasses, vines, bark and clay.

Lemurs at the DLC are maintained in accordance with the U.S. Department of Agriculture regulations and the National Institutes of Health Guide for the Care and Use of Laboratory Animals. The research protocols for this study were approved by the Institutional Animal Care and Use Committee of Duke University (protocol number A171-09-06).

### Study design and sampling

We collected faeces from the sifakas, all of which were individually identifiable via distinguishing markings, at eight time points between July 2015—March 2016 (Fig. 1). For Study 1, we sampled all 31 sifakas once in midsummer and once in midwinter, when their diets were consistently supplemented with diverse blends or single species, respectively. For Study 2, we focused on 11 sifakas that lacked year-round forest access, including from the three social groups that were always denied forest access and from the group that gained forest access only in the summer. These subjects were sampled at six additional times, including 1–2 days before, 2–4 days after and 1 week after the abrupt transitions in fall (i.e., from diverse blends to single species in October) and spring (i.e., from single species to diverse blends in March). We collected samples in the morning (6:30–10:30 H), post voiding, immediately placed them in sterile tubes on ice and stored them at −80 °C within 2 hours of collection.

### Sequencing and bioinformatics

We extracted genomic DNA from faeces using the MoBio Powersoil DNA Isolation Kit (Carlsbad, California, USA) and sent aliquots to Argonne National Laboratory (Lemont, Illinois, USA) for sequencing of the v4 region of the 16S rRNA gene using established methods^57,58^. Sequence data are available in the NCBI Sequence Read Archive under accession numbers SRP158783.

We processed sequences using the Quantitative Insights into Microbial Ecology (QIIME) package (v1.9.1)^57^, using a published analytical workflow^58^. We retained samples that were sequenced to a depth of 10,000 reads and ultimately discarded two samples from downstream analyses. We picked Operational Taxonomic Units (OTUs) using the *de novo* UClust method and based on 97% sequence similarity. OTU taxonomy was assigned using the Greengenes database (v13_8). We used OTUs to calculate alpha-diversity measures, including Good’s Coverage, Chao1, Shannon and Faith’s Phylogenetic Diversity (PD) indices. Good’s Coverage, which estimates what percent of the total number of OTUs in the original community are represented by the sequencing effort, was >95% for all samples. Chao1 captures OTU richness, PD accounts for the relatedness between OTUs, and the Shannon index reflects community evenness^39^. We likewise used OTUs to calculate beta diversity, including unweighted and weighted UniFrac distances. UniFrac distances measure the phylogenetic dissimilarity between pairs of samples, with unweighted measures relying on the presence of OTUs and weighted measures taking into account their relative abundance^40^.

### Nuclear Magnetic Resonance (NMR) spectroscopy

We analysed the colonic metabolome via NMR spectroscopy in a subset of 35 samples from Study 1 using previously established methods^59^ optimized for lemurs^12^. These samples were equally split between midsummer and midwinter, and represented sifakas that gained or were denied forest access. We specifically targeted concentrations of three SCFAs, notably acetate, propionate and butyrate. Spectra were acquired at 600 MHz using a standard Nuclear Overhauser Effect Spectroscopy (NOESY) preset experiment. SCFA concentrations are available in the Supplementary Material (S5).

### Statistical analyses

To assess the influence of foliage diversity and forest access on alpha diversity and SCFA concentrations in Study 1, we implemented Linear Mixed Models (LMM), using the glmmADMB package (version 0.8.3.3^60^) in Rstudio (version 0.99.902^61^). For alpha diversity, we ran three models in which the dependent variable was the Chao1, Shannon or PD index. For SCFAs, we ran three models in which the dependent variable was acetate, propionate or butyrate concentration. We included the sifaka’s identity as a random term and, as explanatory variables, we included foliage condition (two classes: diverse blends or single species), forest access (two classes: yes or no) and their interaction. We considered the sifakas to have forest access if they could semi free-range at any time during the year, even if they were not concurrently in their forested enclosure at the time of sampling. We reran these models including sex as a third explanatory variable and the individual nested within its social group as our random term; however, sex was always an insignificant predictor and neither variable changed the results. We ultimately report the values from the simplified models.

For Study 2, we computed three suites of LMMs on alpha-diversity metrics. In all suites, we ran three models, one for each diversity metric, but we retained only the 11 sifakas that were sampled during the foliage transitions. In the first suite, we used foliage condition as an explanatory variable, but to boost power, we collapsed ‘duplicate’ sampling points into the following four categories: (1) diverse blends (summer and pre-fall transition), (2) single species (winter and pre-spring transition), and (3) the fall and (4) spring transitions (each transition sampled at 2–4 days and 1-week post abrupt foliage switches). In the second and third suites, we retained only the samples from the fall or spring transition (pre transition, 2–4 days and 1-week post abrupt foliage switches), to examine if alpha diversity changed across these one-week periods. For all LMMs, we retained the individual sifaka as a random term. We re-computed all models with sex as an additional explanatory variable and, when possible, the individual nested within its group as a random term; however, as with the larger models above, sex was always an insignificant predictor and neither variable changed the results. We report on findings from the simplified models.

To assess the influence of foliage quality on beta diversity, we calculated pairwise comparisons of unweighted and weighted UniFrac distances using QIIME and Bonferonni-corrected *t*-tests. For Study 1, we compared pairwise distances within and between sifakas that gained or were denied forest access, and that received diverse blends or single species. For Study 2, we compared pairwise distances within and between sifakas during periods of consistent foliage supplements vs. during week-long transitions by collapsing sampling points in the same four categories as earlier: (1) diverse blends and (2) single species, and (3) the fall and (4) spring transitions.

We used Linear Discriminate Analysis Effect Size (LEfSe)^41^ to determine which OTUs varied with foliage condition. Because one cannot examine the interaction between explanatory variables with LEfSe, we examined the influence of foliage condition in Study 1, but not of concurrent forest access. For Study 2, we computed LEfSe to compare transition periods to those when the sifakas consistently received diverse blends or single species. We then used LEfSe analysis to compare communities within the fall and spring transitions, with the pre-transition samples being compared to the other sampling points. To be conservative in our interpretation of LEfSe results, we additionally applied the Benjamini and Hochberg correction factor for multiple testing^62^ using the p.adjust command in RStudio. We include both the uncorrected and corrected results in the Supplemental Material (S2).

To identify which microbes correlated with SCFA concentrations, we computed Kendall’s *tau* correlations using the cor.test function in Rstudio. To avoid spurious correlations, we retained a subset of 38 OTUs that represented either major taxa (i.e., minimally 1% of an individual’s GMB, on average) or varied by foliage supplements (as identified by LEfSe). The results from the full Kendall’s *tau* correlations are presented in the Supplemental Material (S3).

## Electronic supplementary material

S1 Stacked Bar Charts

S2 Full LEfSe Results

S3 SCFA-Microbe Correlations

S4 Dietary Information

S5 SCFA Concentrations

## Data Availability

All sequence data are available in the NCBI Sequence Read Archive under accession numbers SRP158783. SCFA concentrations and associated host metadata are available in the Supplementary Material (S5).
