# Supplementary material for: The critical role of dietary foliage in maintaining the gut microbiome and metabolome of folivorous sifakas
Source: Sci Rep. 2018 Sep 27;8:14482. doi: 10.1038/s41598-018-32759-7 (PMC6160417; doi:10.1038/s41598-018-32759-7)
Supplement: Supplementary file 1 — S1 Stacked Bar Charts [file 41598_2018_32759_MOESM1_ESM.docx]

The critical role of dietary foliage in maintaining the gut microbiome and metabolome of folivorous sifakas

Lydia K Greene, Erin A McKenney, Thomas M O’Connell, & Christine M Drea

Supplementary Information

S1. Stacked bar charts of taxonomic abundance.

**Study 1**

Figure S1.1. Stacked bar charts (y axis) for the subjects of Study 1 (x axis) depicting the most abundant taxa in the sifaka gut microbiome when individuals consumed the diverse blend versus single species foliage. Included here are all the microbial OTUs that accounted for > 1%, on average, of sequences across samples obtained during Study 1. Microbial phyla are bolded and represented by colour families, with individual genera represented by unique colours. ‘Unassigned’ refers to all microbes that could not be assigned to a domain of life; ‘Other’ refers to the sum of all other OTUs that each accounted for < 1%, on average, of sequences across samples obtained during Study 1.

**Study 2**

Figure S1.2. Stacked bar charts (y axis) for the subjects of Study 2 (x axis) depicting the most abundant taxa in the sifaka gut microbiome when individuals consumed the diverse blend versus single species foliage, and during the fall and spring foliage transitions. Included here are all the microbial OTUs that accounted for > 1%, on average, of sequences across samples obtained during Study 2. Microbial phyla are bolded and represented by colour families, with individual genera represented by unique colours. ‘Unassigned’ refers to all microbes that could not be assigned to a domain of life; ‘Other’ refers to the sum of all other OTUs that each account for < 1%, on average, of sequences across samples obtained during Study 2.

Figure S1.3 Stacked bar charts (y axis) for the subjects of Study 2 (x axis) depicting the most abundant taxa in the sifaka gut microbiome during the (top) fall and (bottom) spring foliage transitions. Included here are all the microbial OTUs that accounted for > 1%, on average, of sequences across samples obtained during Study 2. Microbial phyla are bolded and represented by colour families, with individual genera represented by unique colours. ‘Unassigned’ refers to all microbes that could not be assigned to a domain of life; ‘Other’ refers to the sum of all other OTUs that each account for < 1%, on average, of sequences across samples obtained during Study 2.
